# Reducing the futile transportation of out-of-hospital cardiac arrests: a retrospective validation

**DOI:** 10.29045/14784726.2018.09.3.2.1

**Published:** 2018-09-01

**Authors:** Matthew House, Joanne Gray, Peter McMeekin

**Affiliations:** North West Ambulance Service NHS Trust; Northumbria University; Northumbria University

**Keywords:** out-of-hospital cardiac arrest, prediction tool, resuscitation

## Abstract

**Objectives:**

The primary aim was to measure the predictive value of a termination of resuscitation guideline that allows for pre-hospital termination of adult cardiac arrests of presumed cardiac aetiology where the patient did not present in a shockable cardiac rhythm and did not achieve return of spontaneous circulation on-scene. The secondary objective was to compare the effectiveness of that guideline with existing basic life support and advanced life support guidelines.

**Methods:**

A retrospective review of 2139 adult out-of-hospital primary cardiac arrest patients transported to hospital by a single ambulance trust during a 12-month period between 1 April 2014 and 31 March 2015.

**Results:**

Application of the new guideline identified 832 for termination, from which three (0.4%) survived, resulting in a specificity of 99.1% (95% CI: 97.4% to 99.8%), PPV of 99.6% (95% CI: 99% to 99.9%), sensitivity of 46.5% (95% CI: 44.1% to 48.8%) and NPV of 25.6% (95% CI: 23.2% to 28.1%). The transport rate was 60.7%, compared to 72.8% for the basic life support guideline and 95.2% for the advanced life support guideline.

**Conclusions:**

Within the tested cohort, a reduction of 39.3% in transport of adult out-of-hospital primary cardiac arrest of presumed cardiac aetiology could have been achieved if using a termination of resuscitation guideline that allows for termination on-scene when the patient presented in a non-shockable rhythm and there has been no return of spontaneous circulation. These guidelines require prospective validation, but may identify more futile transportations than other previously validated guidelines.

## Introduction

The National Health Service is under pressure to reduce spending, while improving efficiency, and early decisions which reduce the need for unnecessary care are in great demand (NHS England, Public Health England, Health Education England, Monitor, Care Quality Commission, & NHS Trust Development Authority, 2014). During 2016–2017 it was reported that approximately 30,829 out-of-hospital cardiac arrests (OHCA) in England were treated by ambulance services, resulting in a survival rate of only 8.8% (Kay, 2018). The transport of patients with minimal chances of survival represents an ineffective use of both ambulance and emergency department resources (Bonnin, Pepe, Kimball, & Clark, 1993; Cheung, Morrison, & Verbeek, 2001). Currently, UK ambulance guidelines (Joint Royal Colleges Ambulance Liaison Committee & Association of Ambulance Chief Executives, 2016) allow for the termination of resuscitation (TOR) on-scene only when the patient has an asystolic cardiac rhythm following 20 minutes of advanced life support (ALS), and provided drowning, hypothermia poisoning/overdose and pregnancy are not suspected. All other patients, including those with persistent pulseless electrical activity (PEA), should be transported to hospital, unless senior clinical advice is sought. The Resuscitation Council (UK) recognises that the survival to discharge rate for PEA is very low, but suggests that evidence is unclear as to when to stop a resuscitation where PEA persists (Lloyd, 2015).

Previous studies have identified predictors of unsuccessful pre-hospital resuscitation (Morrison et al., 2007; Verbeek et al., 2002). A basic life support (BLS) guideline allows for TOR when there is no return of spontaneous circulation (ROSC), no shocks are administered at any time and the arrest is not witnessed by emergency medical service (EMS) personnel. The ALS guideline adds the conditions that there was no bystander CPR and the arrest was not witnessed by bystanders. These guidelines have been independently validated. However, it has been shown that they are not universal. One EMS system showed survival rates of 1.7% among those predicted not to survive, when the BLS guideline was applied retrospectively, and 4.9% when the ALS guideline was applied (Chiang et al., 2015). Moreover, the rules were not derived in systems that allow for TOR of asystolic arrests and so may not maximise the potential for TOR in these systems. Previously a guideline was derived, which considered those patients currently transported to hospital, and that allows for TOR where the patient does not present with an initial shockable rhythm and does not attain ROSC on-scene (House, Jackson, Dinning, & McMeekin, 2017). As this guideline is of use predominantly where there is persistent PEA, it will be referred to as the PEA guideline. This study sought to validate retrospectively the PEA guideline and to compare its effectiveness with two existing guidelines. Table 1 shows the respective components of each guideline.

**Table 1.** Guideline components.

	Adult only	Presumed cardiac aetiology	No ROSC	No initial shockable rhythm	No shock at any time	Not EMS-witnessed	Not bystander-witnessed	No bystander CPR
**BLS**	✓	✓	✓		✓	✓		
**ALS**	✓	✓	✓		✓	✓	✓	✓
**PEA**	✓	✓	✓	✓				

## Methods

### Study design

This study was a retrospective review of all cases of OHCA transported to hospital by a single ambulance trust during a 12-month period between 1 April 2014 and 31 March 2015. The objective was to measure the predictive value of the decision rule that allows for pre-hospital termination of adult cardiac arrests of presumed cardiac aetiology where the patient did not present in a shockable cardiac rhythm and did not achieve ROSC on-scene. The secondary objective was to compare the effectiveness of this decision rule with existing BLS and ALS guidelines.

### Study setting and population

The data for this study were taken from a large UK Ambulance Trust (the Trust), covering both large urban centres and remote rural areas. It covers a population of 7 million people across a geographical area of approximately 5400 square miles. The Trust has a single tier, combined technician (BLS) and paramedic (ALS) staff. It allows TOR in line with current UK ambulance guidelines (Joint Royal Colleges Ambulance Liaison Committee & Association of Ambulance Chief Executives, 2016). All other OHCA must be transported to hospital.

The data were collected by trained auditors of the Trust’s Governance Department from patient report forms (PRFs) completed by ambulance clinicians following every patient contact, as well as hospital records of patient outcomes. The study included all adult OHCA of presumed cardiac aetiology who were transported to hospital. Patients were excluded from the study if no resuscitation was attempted (i.e. death was diagnosed due to presence of rigor mortis, decomposition and so on, in accordance with present Trust guidelines); if they were under 18 years old; if the arrest was not presumed to have been of cardiac origin (e.g. trauma, drowning or drug overdose); if the resuscitation attempt was terminated under current TOR guidelines; or if their outcome was unknown (two hospitals did not provide follow-up data).

For this study, the three TOR guidelines were applied to cases within the database. We compared the TOR status of each patient as recommended by each guideline and compared this with the actual survival status of patients. We were then able to estimate the transport rate predicted by each of the guidelines for this cohort. We also assessed the sensitivity and specificity of each guideline, and their performance accuracy.

### Data analysis

Statistical analysis was performed using IBM SPSS Statistics 22. For the purposes of analysing the data, we considered death as the positive outcome. Rather than predict survival, this approach attempts to predict death. The resultant guideline would recommend termination for patients who have no hope of survival despite continued resuscitation. 

## Results

Between 1 April 2014 and 31 March 2015, 3920 OHCA were attended by ambulance clinicians in the Trust. Of these, 1781 were excluded from the study. Therefore a total of 2139 patients met the inclusion criteria for initial analysis (see Figure 1). The mean age for the patient group was 69.6 (sd 15.7) years and 64.2% (n = 1373) were male.

**Figure:**
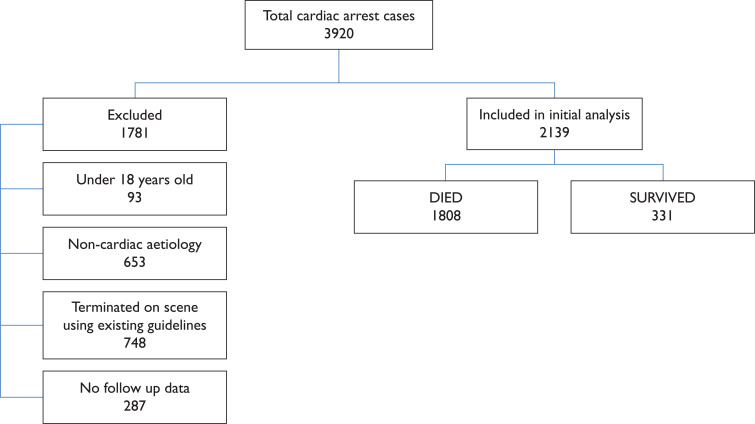
Figure 1. Patient inclusion.

Characteristics of patients were gathered from PRFs. Table 2 describes the out-of-hospital characteristics of eligible patients. Where data were missing, or recorded as ’other’, they were listed as ‘unknown’ for the purposes of analysis.

**Table 2.** Out-of-hospital characteristics of patients.

Characteristic	Number	% of total	Survival within group (%)
Bystander-witnessed	1082	50.5	14.2
EMS-witnessed	376	17.6	30.3
Bystander CPR	1253	68.3	14.7
Shockable rhythm	704	32.9	36.2
Defibrillated	930	43.5	26.6
ROSC	1007	47.1	31.7
Survival to discharge	331	15.4	

Table 3 shows the results of applying the three guidelines to this cohort of patients. It was necessary to exclude 24 patients from the application of the PEA guideline and 11 from the ALS guideline due to incomplete data. Out of 2115 patients with all necessary data, the PEA guideline recommended termination for 832 (39.3%) and transportation for 1283 (60.7%). Of the 832 patients recommended for termination, three (0.4%) survived to hospital discharge. Of those recommended for transport, 328 (25.6%) survived.

**Table 3.** Results.

Observed	PEA guideline		BLS guideline		ALS guideline	
	Transport	Terminate	Transport	Terminate	Transport	Terminate
Died	955 (74.4%)	829 (99.6%)	829 (99.6%)	1226 (78.7%)	582 (100%)	1695 (83.7%)	102 (100%)
% within total	45.2%	39.2%	57.3%	27.2%	79.7%	4.8%
Survived	328 (25.6%)	3 (0.4%)	331 (21.3%)	0	331 (16.3%)	0
% within total	15.5%	0.1%	15.5%	0	15.6%	0
Total	1283	832	1557	582	2026	102

The PEA guideline recommended termination for 829 (46.5%) of patients who died. Of the 832 patients for whom the PEA guideline recommended termination, 829 (99.6%) died and three (0.4%) survived. The transportation rate using the PEA guideline was 60.7%.

The existing BLS and ALS guidelines were applied to the data to compare results. Applying the BLS guideline to the dataset resulted in 1557 (72.9%) patients being recommended for transport. Applying the ALS guideline criteria to the dataset resulted in 2026 (95.2%) patients recommended for transport.

Table 4 describes the characteristics of each guideline. Specificity refers to the guideline’s ability to identify patients who should be transported, and who survive. This is calculated by the equation:

**Table 4.** Characteristics of guidelines.

	PEA guideline % (95% CI)	BLS guideline % (95% CI)	ALS Guideline % (95% CI)
Specificity	99.1 (97.4–99.8)	100 (98.9–100)	100 (98.6–100)
Sensitivity	46.5 (44.1–48.8)	24.4 (22.6–26.1)	4.7 (3.9–5.8)
PPV	99.6 (99.0–99.9)	100 (99.3–100)	100 (95.5–100)
NPV	25.6 (23.2–28.1)	15.5 (14.0–17.1)	14.0 (12.7–15.5)
Transport rate	60.7	72.8	95.2

Specificity = True negatives/

(True negatives + False positives)

Sensitivity refers to a guideline’s ability to identify correctly those patients whose resuscitation attempt should be terminated. This is calculated by the equation:

Sensitivity = True positives /

(True positives + False negatives)

Positive predictive value (PPV) establishes how likely it is that a patient will not survive, when the guideline recommends termination. It is calculated by the equation:

Positive Predictive Value = True positives /

(True positives + False positives)

Negative predictive value (NPV) determines how likely it is that a patient will survive when the guideline recommends transport. It is calculated by the equation:

Negative Predictive Value = True negatives /

(True negatives + False negatives)

The NPV is a good indicator for the effectiveness of any TOR guideline. A high NPV indicates that fewer futile attempts have been categorised as survivable. A high PPV, by contrast, indicates that there are few unexpected survivors in the group predicted to die.

Ideally a TOR guideline would recommend termination for all those patients who will not survive to hospital discharge, as the aim of the guideline is to reduce futile transportation. As such the sensitivity of the guideline is an important characteristic. It will indicate how effective the guideline is at reducing the number of futile transportations.

## Discussion

The issue of TOR in OHCA is important, requiring the balance between the need to achieve ROSC and survival wherever possible against the unnecessary use of limited pre-hospital and hospital resources when transporting patients who have no chance of survival. The decision to transport a patient with OHCA increases the risk to the ambulance clinicians, who are required to transport under emergency blue-light conditions (Kahn, Pirrallo, & Kuhn, 2001). Moreover, transport reduces the availability of that resource to other patients with potentially treatable conditions. Once the patient in refractory cardiac arrest arrives at the receiving hospital, hospital clinicians are required to attend and so are unavailable to other patients for a period of time.

Although ideally TOR should be able to predict patients who have no chance of survival, rather than a low chance of survival, the literature shows TOR decision rules with unexpected survival rates of 0–1% (Kajino et al., 2013; Morrison, Verbeek, Zhan, Kiss, & Allan, 2009; Richman, Vadeboncoeur, Chikani, Clark, & Bobrow, 2008). Survival of <1% has been acknowledged by many as the working definition of medical futility (Morrison et al., 2006; Ong, Jaffey, Stiell, Nesbitt, & OPALS Study Group, 2006; Verbeek et al., 2002). Within this cohort of patients, none of the three guidelines produced unexpected survivors above this 1% threshold. Application of the BLS and ALS guidelines resulted in no unexpected survivors, and application of the PEA guideline resulted in three (0.4%). The first was a 43-year-old male, who had a bystander witnessed cardiac arrest in a public place. Although cannulation was successful, it is recorded that he received no adrenaline. This is inconsistent with guidelines for a patient in cardiac arrest, but the data collected do not explain why this should be the case.

The second patient was a 64-year-old male. The call to scene interval for this patient was nine minutes, the on-scene interval was 57 minutes and the transport interval was seven minutes. This patient suffered an unwitnessed cardiac arrest at home. Bystander CPR was performed. The patient is reported to be asystolic on arrival of the ambulance. He received endotracheal intubation, intravenous access and adrenaline. He was defibrillated three times, the first being reported as 26 minutes after the ambulance arrived on-scene, but was reported not to gain ROSC on-scene.

The final unexpected survivor was a 63-year-old female, who suffered a crew-witnessed cardiac arrest in the ambulance. The presenting rhythm was PEA. The patient was intubated and cannulated. She received adrenaline, but was not defibrillated at any point.

All three guidelines were therefore able to identify potentially survivable resuscitation attempts as previously defined. However, the aim of a TOR guideline is to reduce the number of futile transportations. The new guideline had greater sensitivity than either the BLS or ALS guidelines, and therefore recommended fewer futile transports.

### Limitations

There are several limitations to this study which must be mentioned. Firstly, the database was examined through a secondary analysis of the TOR clinical decision rule rather than prospectively, so there are potential limitations with data integrity and validity. Two of the receiving hospitals in the Trust’s locality did not share data on survival. Also, 24 (1.1%) cases did not record either initial cardiac rhythm or ROSC, so could not be included in the results. It is unknown whether the inclusion of these missing data would put the rule over the <1% futility limit, so the results must be viewed with caution.

The retrospective nature of the study also failed to determine whether paramedics in the field would be able to apply the rule correctly. This means any decision rule we developed conforms only to level 4 of the hierarchy of evidence for decision rules (McGinn et al., 2000). Therefore they would need further prospective evaluation before they are applied clinically. However, as para-medics within the Trust have been successfully applying the existing TOR guideline for over 10 years, and regularly follow clinical decision rules relating to other conditions, this is not considered to be prohibitive. Nevertheless, details of any guideline would need to be addressed before implementation. This would include details such as the required length of resuscitation attempt, before deeming that ROSC had not been achieved, and so on.

Paramedic attitudes and human factors may also have influenced outcomes. A clinician’s perception of the futility of a resuscitation attempt has been shown to affect the duration of that attempt (Bradley et al., 2017). It may be that other elements of the attempt are also affected, though such questions are beyond the scope of this study.

## Conclusion

This study was a retrospective review of adult OHCAs of presumed cardiac aetiology, which were transported to hospital. It sought to evaluate the performance of a TOR guideline that allows for TOR where a patient does not present with an initial shockable rhythm and does not attain ROSC on-scene; and to compare the effectiveness of that guideline with two existing guidelines. All three guidelines were able to recognise potential survivors to within previously agreed limits of futility. However, the proposed TOR guideline identified more futile transportations. The application of this guideline would have reduced futile transportation of those patients currently transported by 39.3%. Further work is required to validate this rule prospectively, before it can be applied in clinical settings.

## Acknowledgements

Matthew House is the primary author of the manuscript and had full access to all of the data in the study and takes responsibility for the integrity of the data and the accuracy of the data analysis. He would like to acknowledge Northumbria University for supporting this study, and the North West Ambulance Service NHS Trust for providing access to their cardiac arrest database.

## Conflict of interest

None declared.

## Ethics

Approval received from the institutional research ethics board, the NHS Health Research Authority and the University Research Ethics Board.

## Funding

This research was funded by the North West Ambulance Service NHS Trust and Northumbria University.
